# Safety and short-term outcomes of laparoscopic surgery for advanced gastric cancer after neoadjuvant immunotherapy: A retrospective cohort study

**DOI:** 10.3389/fimmu.2022.1078196

**Published:** 2022-12-08

**Authors:** Jin Su, Weihong Guo, Zhian Chen, Lingzhi Wang, Hao Liu, Liying Zhao, Tian Lin, Fengping Li, Xinyuan Mao, Huilin Huang, Jiang Yu, Guoxin Li, Yanfeng Hu

**Affiliations:** ^1^ Department of General Surgery & Guangdong Provincial Key Laboratory of Precision Medicine for Gastrointestinal Tumor, Nanfang Hospital, The First School of Clinical Medicine, Southern Medical University, Guangzhou, China; ^2^ Department of General Surgery, Zhuzhou Hospital affiliated to Xiangya School of Medicine, Central South University, Zhuzhou, China

**Keywords:** gastric cancer, neoadjuvant immunotherapy, laparoscopic surgery, safety, outcome

## Abstract

**Background:**

Immune checkpoint inhibitors (ICIs) have been increasingly used for the treatment of advanced gastric cancer (AGC). However, the safety and the short-term outcomes of laparoscopic gastrectomy for patients with AGC after neoadjuvant immunotherapy (NAI) remain unknown.

**Methods:**

We retrospectively analyzed the patients with AGC who underwent laparoscopic surgery after neoadjuvant therapy between 1 January 2019 and 31 October 2021. We further compared the differences in postoperative complications, overall response rate, adverse events, surgical parameters, and postoperative recovery between two cohorts: the NAI group (NAI plus chemotherapy) and the neoadjuvant chemotherapy (NAC) group. Multivariable regression analyses were used to determine the risk factors for the overall response rate.

**Results:**

Overall, 80 patients were enrolled, of whom 30 cases were included in the NAI cohort and 50 were included in the NAC cohort. The overall rate of postoperative complications was 30.0% in both groups (*p =* 1.000). The overall response rate was 70.0% in the NAI cohort and 40% in the NAC cohort (*p =* 0.012). The adverse effects were found in 16 cases (53.3%) of the NAI cohort and 23 cases (46.0%) of the NAC cohort (*p =* 0.645). There was no statistical difference in intraoperative bleeding (50 ml vs. 50 ml, *p =* 0.983), operation time (320.9 min vs. 303.5 min, *p =* 0.382), dissected lymph node count (43.5 vs. 40.0, *p =* 0.364), first postoperative anal aerofluxus (3 days vs. 3 days, *p =* 0.091), first liquid diet (4 days vs. 5 days, *p =* 0.213), and postoperative length of stay in the hospital (8 days vs. 7 days, *p =* 0.508) between the two groups. NAI was estimated to be the independent protective factor [odds ratio (OR) 4.931, 95% confidence interval (CI) (1.385–17.559), *p =* 0.014] for odds to overall response rate, whereas vessel invasion was found to be the significant risk factor [OR 0.113, 95% CI (0.027–0.475), *p =* 0.003].

**Conclusions:**

Laparoscopic surgery after NAI combined with chemotherapy is a safe therapeutic choice for AGC and may bring better short-term outcomes due to a higher overall response rate.

## Introduction

Gastric cancer is the fourth leading cause of cancer-related death worldwide, with over 1 million new cases annually (1). Surgical procedure still plays a pivotal role in the treatment of gastric cancer. Since the 21st century, there have been gradual changes in the therapeutic strategies of gastric cancer, and laparoscopic surgery has been broadly applied in this field. The CLASS-01 trial of the Chinese Laparoscopic Gastrointestinal Surgery Study group (2) confirmed that laparoscopic surgery is safe and feasible for local advanced gastric cancer (AGC), and patients treated by this surgery could achieve better recovery postoperatively. For AGC patients undergoing the surgical procedure after neoadjuvant chemotherapy (NAC), the Idea, Development, Exploration, Assessment, Long-term (IDEAL) study (3) showed that laparoscopic surgery is safer compared to open surgery, accompanied by better tolerance of those patients to postoperative adjuvant chemotherapy. Moreover, laparoscopic surgery would lead to less intraoperative bleeding, shorter postoperative length of stay in the hospital, as well as similar overall survival (OS) and 5-year disease-free survival (DFS) rates (4).

Nowadays, immune checkpoint inhibitors (ICIs) have been confirmed to be beneficial in inducing anti-tumor immune responses, which are recommended as an important therapeutic approach for AGC (5). An increasing number of trials have been implemented on the application of immunotherapy combined with conventional chemotherapy as a first-line adjuvant therapy for unresectable gastric or gastroesophageal junction cancer, with the achievement of positive results. According to these reports, when the combined positive score (CPS) is greater than 5 or 10, immunotherapy-based combined therapy can prolong the OS and DFS when compared with chemotherapy alone (6, 7). Therefore, one ICI that targets programmed death 1 (PD-1), nivolumab, as a new promising therapeutic choice for advanced-stage cancers, has been approved by the Food and Drug Administration (FDA) in 2021 for the first-line treatment of AGC or metastatic gastric cancer (8).

As for the ICIs that target PD-1/programmed death ligand 1 (PD-L1), there are still some adverse effects in the clinical practice, which occur generally in delayed periods and tend to be autoimmune specialty compared with chemotherapy. The top 5 adverse events are rash, colitis, pneumonia, elevated transaminases, and hypothyroidism (9). As evidenced by preliminary clinical trials, the incidence of adverse effects of single-agent ICIs is 15% to 90%, while the overall incidence of adverse effects of PD-1/PD-L1 inhibitors is approximately 30%, of which the incidence of grades 3/4 adverse effects is 9%, and that of serious adverse events requiring immunosuppressive therapy or discontinuation of immunotherapy ranges from 0.5% to 13% (10, 11). For unresectable gastric or gastroesophageal junction cancer, the incidence of grades 3/4 adverse effects of chemotherapy combined with immunotherapy ranges from 59% to 72%, and 2% of patients died due to severe adverse effects (6, 7).

At present, the clinical application of immunotherapy has aroused a certain degree of interest, while the safety and the short-term outcomes of laparoscopic surgery for AGS after neoadjuvant immunotherapy (NAI) remain unclear. It remains unknown whether the activation of the immune system aggravates inflammatory stress after NAI for AGC, and whether the superimposed adverse effects of NAI and conventional chemotherapy would delay the time of laparoscopic surgery and aggravate postoperative complications (e.g., gastrointestinal inflammation, edema, or post-operational pulmonary inflammation). These are a series of practical clinical questions that have not been well answered according to the current research. Therefore, the present study was carried out with the primary endpoint to determine the safety and short-term outcomes of laparoscopic surgery for AGC after NAI combined with chemotherapy compared with NAC alone.

## Methods

### Study design

The clinical data of enrolled patients were collected from Nanfang Hospital of Southern Medical University between 1 January 2019 and 31 October 2021. The inclusion criteria were as follows: (1) patients aged between 18 and 80 years; (2) patients with the Eastern Cooperative Oncology Group (ECOG) score of <2 points before treatment; (3) patients without serious cardiopulmonary disorders, other comorbidity, and serious coagulation dysfunction; (4) patients without previous treatment and with the diagnosis of gastric cancer by endoscopic and pathological methods; and (5) patients with preoperative clinical tumor stage of cT1-2N+M0, cT3-4bNanyM0. Furthermore, patients diagnosed with limited metastasis and who have the possibility of undergoing radical resection after neoadjuvant therapy (NAT), which was evaluated by multidiscipline discussion, were enrolled in our study. The exclusion criteria were as follows: patients who were suspected of or confirmed with multiple distant metastases and recurrent gastric cancer, combined with other malignant tumors, co-existing with other organ insufficiency, and those who were in the acute phase of certain infections. Patients who met the criteria for inclusion and exclusion were enrolled and assigned into two groups [NAI group (NAI plus chemotherapy) and NAC group] randomly to eliminate selection bias. Meanwhile, all patients received consecutive NAT before laparoscopic gastrectomy.

### Outcome measurements

The cTNM staging and ypTNM staging were conducted following the 8th edition of the American Joint Committee on Cancer (AJCC) staging system (12). Contrast-enhanced computed tomography was used for pre-therapeutic clinical TNM staging assessment. Standard radical gastrectomy with D2 lymphadenectomy was performed for each patient by following the Japanese Gastric Cancer Association’s gastric cancer treatment guidelines (13). The primary outcomes were safety, which was defined as the rate of surgical complications within 1 month postoperatively and was evaluated by the Clavien–Dindo classification system (14), and short-term outcome, which was defined as postoperative overall response rate based on the Becker criteria (15). In addition, the secondary outcomes were adverse events [assessed according to the National Cancer Institute Common Terminology Criteria for Adverse Events (CTCAE 4.0)], surgical parameters (intraoperative bleeding, operation time, and dissected lymph node count), and postoperative recovery (including first anal aerofluxus, first liquid diet, and postoperative length of stay in the hospital, which were assessed within 2 weeks postoperatively).

### Therapeutic regimens of NAC and NAI

The patients in the NAC group received two to eight cycles (70.0% received three to four cycles) of consecutive treatment before surgery, and the main treatment regimens include CapeOX (oxaliplatin at 130 mg/m^2^ on day 1 and capecitabine at 1,000 mg/m^2^ twice daily from days 1 to 14, 21 days per cycle), FOLFOX (oxaliplatin at 85 mg/m^2^ on day 1, fluorouracil at 400 mg/m^2^ on day 1 and 2,400 mg/m^2^ on days 1 and 2 through continuous infusion, and leucovorin at 400 mg/m^2^ on day 1, 2 weeks per cycle), and FLOT (docetaxel at 50 mg/m^2^ on day 1, oxaliplatin at 85 mg/m^2^ on day 1, fluorouracil at 2,600 mg/m^2^ for 24 h through continuous infusion, and leucovorin at 200 mg/m^2^ on day 1, 21 days per cycle), while patients in the NAI cohort received combined treatment using ICIs based on NAC for two to eight treatment cycles (86.7% received three to four cycles) similarly. The ICIs included pembrolizumab (200 mg), toripalimab (3 mg/kg), carrelizumab (200 mg), and sintilimab (200 mg), all of which were administrated intravenously every 21 days as a cycle.

### Laparoscopic surgery

All patients underwent laparoscopic surgery after NAT. Total or partial gastrectomy combined with D2 lymph node dissection was performed according to the tumor size and location. Specifically, laparoscopic distal gastrectomy was performed for tumors located at the antrum or the distal stomach body. Total gastrectomy was conducted when the tumors were located at the fundus, upper site of the stomach body, and gastroesophageal junction. All operations were completed by experienced chief surgeons. Reconstruction of the alimentary tract was accomplished by Billroth II gastroduodenostomy or Roux-en-Y esophagojejunostomy according to the types of gastrectomy and at the surgeon’s discretion. Combined resection was performed for those with local invasion or limited distant metastasis, and it was estimated that R0 resection could be achieved.

### Statistical analysis

Two groups of populations for analysis were described. The intention-to-treat population was enrolled according to the inclusion and exclusion criteria and was used to calculate the baseline characteristics. The as-treated population excluded the patients who did not complete the full treatment of NAT and laparoscopic gastrectomy. The primary and secondary end points were calculated by the as-treated population.

The SPSS^®^ version 26.0 (IBM, Armonk, New York, USA) software package was used for statistical analysis and data processing. The normality of the distribution of continuous variables of each group was assessed using the Kolmogorov–Smirnov test. The continuous variables were presented as mean (standard deviation, SD) if normally distributed, and as median (interquartile range, IQR) if not. Univariate analysis was conducted to evaluate the differences between all baseline parameters and short-term outcome indicators of each group. The continuous data of each group were evaluated using Student’s *t*-test or Mann–Whitney test for differences. Meanwhile, categorical data were analyzed by *χ* ^2^ test or Fisher’s exact test if appropriate. Univariable and multivariable regression analyses were used to identify factors associated with the overall response rate. Odds ratio (OR) with a 95% confidence interval (CI) was shown. Variables in the univariable analysis were included in the multivariable model when *p* < 0.05. Two-tailed *p* < 0.05 was considered statistically significant.

## Results

### Patient enrollment and baseline clinical data

**Figure 1:**
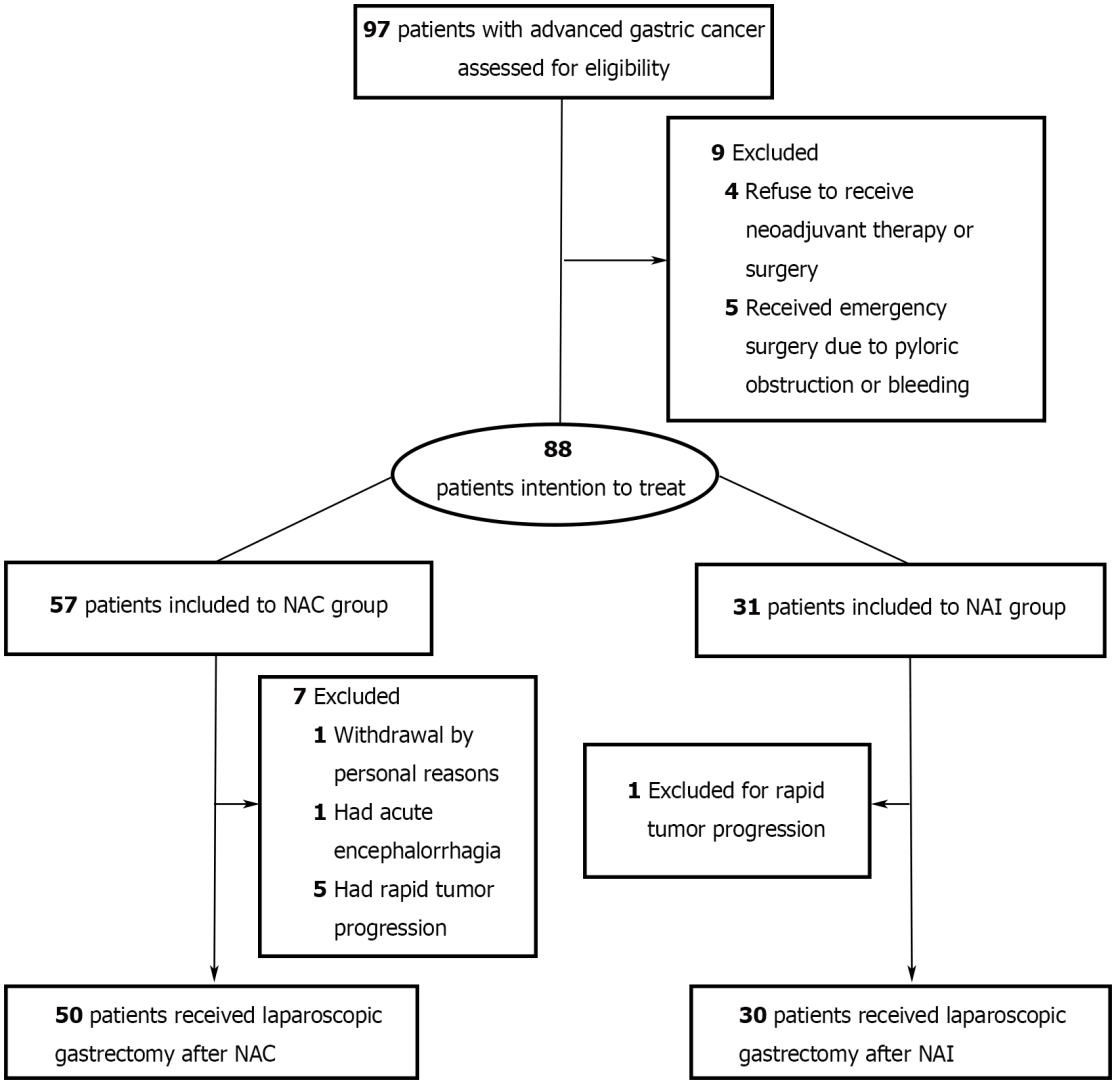
Study flow diagram. NAI, neoadjuvant immunotherapy; NAC, neoadjuvant chemotherapy.

Overall, 88 patients were enrolled and allocated to the NAI group or NAC group (**Figure 1**). No statistically significant difference was found in baseline clinical parameters [sex, age, body mass index (BMI), cTNM stage, and ECOG] between the two cohorts (**Table 1**, all *p >* 0.05). Six patients had rapid tumor progression, one patient suffered from acute encephalorrhagia, and one patient declined surgery; all of these patients were excluded from this study. Finally, 80 patients met the criteria of the as-treated population (**Supplementary Table 1**). There were six cases and four cases with limited metastasis in the NAI group and NAC group, respectively. Intergroup analysis (NAI vs. NAC) revealed no statistical significance in terms of the performance status evaluated by ECOG scores (*p =* 0.800). The percentage of male and female patients in the NAI group was 64.5% and 35.5%, and it was 77.2% and 22.8% in the NAC group. The median (IQR) age of patients was 58 (45–67) years in the NAI cohort and 57 (50–65) years in the NAC cohort, respectively. The mean (SD) BMI in the NAI group and the NAC group was 23.5 (3.6) kg/m^2^ and 22.5 (2.7) kg/m^2^, respectively. Nine (30.0%) of 30 cases from the NAI cohort and 20 (40.0%) of 50 cases from the NAC cohort had tumors located in the upper stomach, while 40.0% were located in the lower site of each cohort.

**Table 1:** Clinical data characteristics of intention-to-treat patients.

Variable	NAC (*n* = 57)	NAI (*n* = 31)	*p-*value^c^
Sex			0.220
Male	44 (77.2)	20 (64.5)	
Female	13 (22.8)	11 (35.5)	
Age (years)			0.785
Median^a^	57 (50–65)	58 (45–67)	
<60	35 (61.4)	17 (54.8)	
60–69	18 (31.6)	11 (35.5)	
≥70	4 (7.0)	3 (9.7)	
BMI (kg/m^2^)			0.220^d^
Mean^b^	22.5 (2.7)	23.5 (3.6)	
Range	15.4–27.7	16.6–29.7	
ECOG			0.800
0	15 (26.3)	7 (22.6)	
1	42 (73.7)	24 (77.4)	
cT stage			0.386
T1–2	3 (5.3)	0	
T3	12 (21.1)	4 (12.9)	
T4A	33 (57.9)	19 (61.3)	
T4B	9 (15.8)	8 (25.8)	
cN stage			1.000
N0	4 (7.0)	2 (6.5)	
N+	53 (93.0)	29 (93.5)	
cM stage			0.156
M0	53 (93.0)	25 (80.6)	
M1	4 (7.0)	6 (19.4)	
cTNM stage			0.198
II	7 (12.3)	1 (3.2)	
III	37 (64.9)	18 (58.1)	
IVA	9 (15.8)	6 (19.4)	
IVB	4 (7.0)	6 (19.4)	

Values in parentheses are percentages. NAC, neoadjuvant chemotherapy; NAI, neoadjuvant immunotherapy. ^a^Values are shown as median (IQR). ^b^Values are shown as mean (SD). ^c^p-value was calculated by χ^2^ test or Fisher’s exact test, except for ^d^Mann–Whitney test.

### Adverse events

The two groups of patients received two to eight cycles of continuous NAT preoperatively. The proportion of patients in the NAI group who received three to four cycles of neoadjuvant therapy is 86.7%, and the proportion is 70.0% in the NAC group (data were not shown, *p =* 0.109). The main regimens in the NAC group included CapeOX, FOLFOX, and FLOT, and the NAI group applied combined therapy using PD-1 inhibitors (carelizumab, pembrolizumab, toripalizumab, and sintilimab) on the basis of the regimens adopted in the NAC group. There was no significant difference in adverse effects of the hematopoietic system and non-hematopoietic system in patients between the NAI group and the NAC group (**Table 2**, 53.3% vs. 46.0%, *p =* 0.645). Most of the adverse effects in the two groups were grades 1/2. The common adverse effects of the hematopoietic system were leukopenia, neutropenia, and anemia, while those of the non-hematopoietic system were nausea, vomiting, and elevated transaminases. In addition, 10.0% and 8.0% of the patients in NAI and NAC groups had grades 3/4 adverse effects, and the adverse events above grade 4 were not observed.

**Table 2:** Adverse events.

Variable^a^	NAC (*n* = 50)	NAI (*n* = 30)	*p-*value^b^
Leukopenia			
Grade 0	41 (82.0)	23 (76.7)	
Grade 1–2	9 (18.0)	6 (20.0)	
Grade 3–4	0	1 (3.3)	
Neutropenia			
Grade 0	41 (82.0)	22 (73.3)	
Grade 1–2	7 (14.0)	8 (26.7)	
Grade 3–4	2 (4.0)	0	
Anemia			
Grade 0	43 (86.0)	26 (86.7)	
Grade 1–2	6 (12.0)	4 (13.3)	
Grade 3–4	1 (2.0)	0	
Thrombocytopenia			
Grade 0	48 (96.0)	23 (76.7)	
Grade 1–2	2 (4.0)	5 (16.7)	
Grade 3–4	0	2 (6.7)	
Nausea			
Grade 0	44 (88.0)	29 (96.7)	
Grade 1–2	6 (12.0)	1 (3.3)	
Grade 3–4	0	0	
Vomiting			
Grade 0	45 (90.0)	29 (96.7)	
Grade 1–2	5 (10.0)	1 (3.3)	
Grade 3–4	0	0	
Diarrhea			
Grade 0	49 (98.0)	30 (100.0)	
Grade 1–2	1 (2.0)	0	
Grade 3–4	0	0	
Fatigue			
Grade 0	49 (98.0)	30 (100.0)	
Grade 1–2	1 (2.0)	0	
Grade 3–4	0	0	
Aminotransferase increased			
Grade 0	47 (94.0)	26 (86.7)	
Grade 1–2	2 (4.0)	4 (13.3)	
Grade 3–4	1 (2.0)	0	
Bilirubin increased			
Grade 0	50 (100.0)	28 (93.3)	
Grade 1–2	0	2 (6.7)	
Grade 3–4	0	0	
Rash			
Grade 0	49 (98.0)	29 (96.7)	
Grade 1–2	1 (2.0)	1 (3.3)	
Grade 3–4	0	0	
Grade 1–2 rate	22 (44.0)	14 (46.7)	1.000
Grade 3–4 rate	4 (8.0)	3 (10.0)	1.000
Overall rate^a^	23 (46.0)	16 (53.3)	0.645

Values in parentheses are percentages. NAC, neoadjuvant chemotherapy; NAI, neoadjuvant immunotherapy. ^a^Multiple adverse effects may occur in one patient. ^b^p-value was calculated by χ^2^ test or Fisher’s exact test.

### Surgical and pathological results

There were no statistically significant differences in surgical indicators in the NAI and NAC cohorts (**Table 3**, all *p >* 0.05), while the median interval in the NAI group from the last NAT to laparoscopic gastrectomy was longer than that of the NAC cohort [36 (30–46) vs. 28 (23–36) days, *p =* 0.001]. However, no cases underwent unplanned procedures or dropped out of subsequent therapy owing to serious adverse effects in the NAI cohort. The rate of total gastrectomy was 53.3% in the NAI cohort and 56.0% in the NAC cohort. Roux-en-Y digestive tract reconstruction was mainly performed in both groups (93.3% vs. 74.0% for NAI and NAC groups). Five (16.7%) and six patients (12.0%) in each cohort underwent combined organ resection. The R0 resection rate of the two cohorts (NAI vs. NAC) was 86.7% and 84.0%. The mean (SD) operation time of the two cohorts was 320.9 (88.4) min and 303.5 (84.6) min (*p =* 0.382). There was no significant difference regarding median (IQR) intraoperative bleeding [50 (50–100) vs. 50 (50–100) ml, *p =* 0.983] and mean (SD) dissected lymph node count [43.5 (14.3) vs. 40.0 (17.8), *p =* 0.364] between the two cohorts. No patients converted to laparotomy in either group. There were no differences in vessel invasion (33.3% vs. 38.0%, *p =* 0.811) and nerve invasion (46.6% vs. 62.0%, *p =* 0.245) between the two cohorts. However, a significant difference was observed regarding tumor regression grade between the two cohorts (*p* < 0.001), and more patients in the NAI cohort obtained grade 1A (33.3% vs. 2.0%). The overall response rate in the NAI cohort was significantly higher than that of the NAC cohort (70.0% vs. 40.0%, *p =* 0.012).

**Table 3:** Surgical, postoperative recovery, and pathological results of two groups.

Variable	NAC (*n* = 50)	NAI (*n* = 30)	*p-*value^c^
Tumor location			0.537
Upper	20 (40.0)	9 (30.0)	
Middle	10 (20.0)	9 (30.0)	
Lower	20 (40.0)	12 (40.0)	
Type of gastrectomy			1.000
Distal	22 (44.0)	14 (46.7)	
Total	28 (56.0)	16 (53.3)	
Type of reconstruction			0.040
Billroth II	13 (26.0)	2 (6.7)	
Roux-en-Y	37 (74.0)	28 (93.3)	
Extent of resection			1.000
R0	42 (84.0)	26 (86.7)	
R1	8 (16.0)	4 (13.3)	
Vessel invasion			0.811
Positive	19 (38.0)	10 (33.3)	
Negative	31 (62.0)	20 (66.7)	
Nerve invasion			0.245
Positive	31 (62.0)	14 (46.6)	
Negative	19 (38.0)	16 (53.4)	
Multiorgan resection (yes)	6 (12.0)	5 (16.7)	0.739
Lymph nodes removed (no.)^b^	40.0 (17.8)	43.5 (14.3)	0.364^e^
Convert to open surgery	0	0	NA
NAT to surgery interval (days)^a^	28 (23–36)	36 (30–46)	0.001^d^
Bleeding (ml)^a^	50 (50–100)	50 (50–100)	0.983^d^
Operation time (min)^b^	303.5 (84.6)	320.9 (88.4)	0.382^e^
First aerofluxus (days)^a^	3 (3)	3 (3-4)	0.091^d^
First liquid diet (days)^a^	5 (3-6)	4 (3-4)	0.213^d^
Postoperative hospital stay (days)^a^	7 (5-9)	8 (6-9)	0.508^d^
ypT stage			0.001
T0	1 (2.0)	11 (36.7)	
T1A	2 (4.0)	0	
T1B	2 (4.0)	0	
T2	2 (4.0)	2 (6.7)	
T3	21 (42.0)	10 (33.3)	
T4A	11 (22.0)	5 (16.7)	
T4B	11 (22.0)	2 (6.7)	
ypN stage			0.193
N0	15 (30.0)	16 (53.3)	
N1	11 (22.0)	6 (20.0)	
N2	8 (16.0)	2 (6.7)	
N3	16 (32.0)	6 (20.0)	
ypM stage			0.416
M0	47 (94.0)	26 (86.7)	
M1	3 (6.0)	4 (13.3)	
ypTNM stage			0.001
0	1 (2.0)	10 (33.3)	
I	5 (10.0)	1 (3.3)	
II	14 (28.0)	6 (20.0)	
III	27 (54.0)	9 (30.0)	
IV	3 (6.0)	4 (13.3)	
Tumor regression grade^f^			< 0.001
1A	1 (2.0)	10 (33.3)	
1B	3 (6.0)	1 (3.3)	
2	16 (32.0)	10 (33.3)	
3	30 (60.0)	9 (30.0)	
Overall response rate	20 (40.0)	21 (70.0)	0.012

Values in parentheses are percentages. NA, not applicable; NAC, neoadjuvant chemotherapy; NAI, neoadjuvant immunotherapy; NAT, neoadjuvant therapy. ^a^Values are shown as median (IQR). ^b^Values are shown as mean (SD). ^c^p-value was calculated by χ^2^ test or Fisher’s exact test, except ^d^Mann–Whitney test and ^e^Student’s t-test. ^f^Tumor regression grade followed by Becker criteria.

### Postoperative complications and recovery

There was no significant difference in the NAI cohort and NAC cohort regarding the overall complications within 30 days postoperatively (**Table 4**, *p =* 1.000). Meanwhile, no statistically significant difference was found in the overall postoperative complications in the NAI cohort of our study when compared with the regimen of using NAC plus laparoscopic gastrectomy and laparoscopic gastrectomy alone as previously reported (**Supplementary Table 2**, *p =* 0.083). Furthermore, there were seven cases (14.0%) and six cases (20.0%) that suffered from pulmonary infection postoperatively in the NAC group and NAI group, respectively. One patient (3.3%) in the NAI cohort suffered from full-thickness wound dehiscence, and five patients (10.0%) in the NAC cohort had anastomotic leakage and underwent reoperation, yet without statistical significance between the two cohorts (all *p >* 0.05). There was one patient in each group who suffered from anastomotic bleeding and underwent conservative treatment and recovered finally. Two cases (6.7%) in the NAI cohort and four cases (8.0%) in the NAC cohort suffered from grades 3/4 complications. No perioperative death was found in either group. There was no difference in the median (IQR) time of the first anal aerofluxus [3 (3–4) vs. 3 (3) days, *p =* 0.091] and the first liquid diet [4 (3–4) vs. 5 (3-6) days, *p =* 0.213] between the two groups. The median (IQR) postoperative length of stay in the hospital of the two groups was 8 (6–9) days and 7 (5–9) days (*p =* 0.508), respectively.

**Table 4:** Postoperative complications.

Variable	NAC (*n* = 50)	NAI (*n* = 30)	*p-*value^b^
Clavien–Dindo classification			
Grade I	1 (2.0)	0	1.000
Grade II	12 (24.0)	8 (26.7)	1.000
Grade IIIa	0	0	NA
Grade IIIb	4 (8.0)	1 (3.3)	0.645
Grade IVa	0	1 (3.3)	0.375
Overall complications^a^	15 (30.0)	9 (30.0)	1.000
Hemorrhage	1 (2.0)	1 (3.3)	1.000
Anastomotic leakage	5 (10.0)	0	0.151
Intestinal obstruction	2 (4.0)	0	0.525
Wound complications	1 (2.0)	1 (3.3)	1.000
Pulmonary infection	7 (14.0)	6 (20.0)	0.539
Thromboembolism	0	1 (3.3)	0.375
Heart failure	0	1 (3.3)	0.375
Reoperation	4 (8.0)	1 (3.3)	0.645

Values in parentheses are percentages. NA, not applicable; NAC, neoadjuvant chemotherapy; NAI, neoadjuvant immunotherapy. ^a^Multiple complications may occur in one patient. ^b^p-value was calculated by χ^2^ test or Fisher’s exact test.

### Multivariable analyses to determine factors associated with the overall response rate

Univariable analyses of the overall response rate (partial response and complete response) in all patients revealed that multiple items were estimated to be the predictors (**Table 5**). NAI was founded to be the only protective factor [OR 4.931, 95% CI (1.385–17.559), *p =* 0.014] for odds to overall response rate, and vessel invasion as the independent risk factor [OR 0.113, 95% CI (0.027–0.475), *p =* 0.003] in the multivariable regression model. Univariable analyses of the overall response rate for the NAI group estimated three variables that were included in the multivariable model (**Table 6**). However, none of them were identified as predictors with statistically significant difference.

**Table 5:** Multivariable regression analysis of risk factors for the overall response rate in all patients.

Variables	Univariable analysis		Multivariable analysis	
	OR (95% CI)	*p-*value	OR (95% CI)	*p-*value
Sex (male vs. female)	1.397 (0.505–3.860)	0.519		
Age (≥65 vs. <65 years)	0.516 (0.197–1.350)	0.178		
BMI (≥25 vs. <25 kg/m^2^)	0.690 (0.249–1.913)	0.476		
Leukopenia (yes vs. no)	0.497 (0.161–1.532)	0.224		
Granulocytopenia (yes vs. no)	0.313 (0.098–0.993)	0.049	0.512 (0.099–2.653)	0.425
Anemia (yes vs. no)	0.494 (0.132–1.843)	0.294		
Thrombocytopenia (yes vs. no)	1.215 (0.301–4.902)	0.784		
NAT regimen (NAI vs. NAC)	3.500 (1.334–9.180)	0.011	4.931 (1.385–17.559)	0.014
Tumor location (distal vs. proximal)	2.605 (1.042–6.512)	0.040	1.941 (0.406–9.283)	0.406
Vessel invasion (yes vs. no)	0.119 (0.041–0.350)	<0.001	0.113 (0.027–0.475)	0.003
Nerve invasion (yes vs. no)	0.221 (0.085–0.573)	0.002	0.308 (0.090–1.056)	0.061
Extent of resection (R1 vs. R0)	0.635 (0.183–2.200)	0.474		
Type of gastrectomy (total vs. distal)	0.315 (0.125–0.791)	0.014	0.262 (0.054–1.266)	0.096
Multiorgan resection (yes vs. no)	0.306 (0.075–1.252)	0.099		
Metastasis (yes vs. no)	0.595 (0.154–2.292)	0.450		

BMI, body mass index; CI, confidence interval; NAC, neoadjuvant chemotherapy; NAI, neoadjuvant immunotherapy; NAT, neoadjuvant therapy; OR, odds ratio.

**Table 6:** Multivariable regression analysis of risk factors for the overall response rate to the NAI group.

Variables	Univariable analysis		Multivariable analysis	
	OR (95% CI)	*p-*value	OR (95% CI)	*p-*value
Sex (male vs. female)	4.000 (0.765–20.920)	0.101		
Age (≥65 vs. <65 years)	0.938 (0.194–4.522)	0.936		
BMI (≥25 vs. <25 kg/m^2^)	1.000 (0.191–5.241)	1.000		
CPS (≥1 vs. <1)	0.531 (0.051–5.553)	0.597		
HER2 (positive vs. negative)	1.231 (0.238–6.358)	0.804		
Leukopenia (yes vs. no)	0.208 (0.035–1.254)	0.087		
Granulocytopenia (yes vs. no)	0.294 (0.053–1.622)	0.160		
Anemia (yes vs. no)	0.368 (0.043–3.141)	0.361		
Thrombocytopenia (yes vs. no)	0.471 (0.081–2.743)	0.402		
Tumor location (distal vs. proximal)	1.818 (0.357–9.272)	0.472		
Vessel invasion (yes vs. no)	0.118 (0.020–0.686)	0.017	0.224 (0.026–1.965)	0.177
Nerve invasion (yes vs. no)	0.143 (0.023–0.877)	0.036	0.162 (0.014–1.877)	0.145
Extent of resection (R1 vs. R0)	0.368 (0.043–3.143)	0.361		
Type of gastrectomy (total vs. distal)	0.455 (0.089–2.318)	0.343		
Multiorgan resection (yes vs. no)	0.211 (0.028–1.573)	0.129		
Metastasis (yes vs. no)	0.132 (0.018–0.936)	0.043	0.074 (0.005–1.169)	0.064

BMI, body mass index; CI, confidence interval; CPS, combined positive score; HER2, human epidermal growth factors receptor 2; NAC, neoadjuvant chemotherapy; NAI, neoadjuvant immunotherapy; OR, odds ratio.

## Discussion

At present, research on the application of immunotherapy in gastric cancer generally focuses on patients with unresectable, metastatic, or recurrent tumors (7, 16, 17). The safety and efficacy of adjuvant immunotherapy have been adequately evaluated for these patients. However, the safety and the short-term efficacy of NAI and subsequent laparoscopic gastrectomy for patients with AGC remain uncertain. Accordingly, the present study was conducted to clarify the above issues of NAI combined with chemotherapy versus conventional NAC for AGC followed by laparoscopic surgery, which was compared in the aspects of postoperative complications, pathological regression, adverse events, and postoperative recovery. Consequently, NAI combined with chemotherapy plus laparoscopic surgery is safe and can achieve better pathological responses for the treatment of patients with AGC.

The accumulated number of clinical trials has confirmed that NAC or perioperative chemotherapy can bring survival benefits to patients with AGC (18, 19). Nowadays, NAC represents a promising approach and has become a standard treatment for AGC worldwide (6, 20). Common NAC regimens for AGC include CapeOX, FOLFOX, SOX, SP, ECF/ECX, FLOT, and DOS, and corresponding incidences of adverse effects were 24%, 33%, 20.8%, 34.4%, 27%, 27%, and 15.2%, respectively. The choice of regimens has regional differences from Eastern to Western countries (21–25). However, it is still unknown whether NAI combined with conventional chemotherapy may increase the incidence of adverse effects, cause unplanned procedures, elevate postoperative complications, and delay postoperative recovery. In early breast cancer and advanced head and neck carcinoma, NAI combined with conventional chemotherapy produced no impact on the occurrence of severe grades 3/4 adverse events and unplanned surgery (26, 27). Our study found that the overall adverse effect rate of NAI combined with conventional chemotherapy and NAC alone was 53.3% and 46.0% for AGC, and the incidence of grades 3/4 adverse effects was 10.0% and 8.0%, respectively, neither of which showed significant statistical difference. In addition, there was no unplanned operation due to serious adverse events in the NAI cohort, although the median interval from the last treatment to surgery in the NAI cohort was longer than that in the NAC group. However, the treatment-related adverse event was similar in the two groups, and there were no patients who dropped out because of serious adverse effects. This delay might be partially attributed to safety concerns due to a poor understanding of ICIs as NAT before laparoscopic surgery. Findings in our study shared similarities to the research results in the breast cancer and head and neck carcinoma mentioned above.

Prior research (28) has documented that postoperative complications of gastric cancer can affect the completion of subsequent comprehensive anti-tumor therapy, suggesting that a higher complication rate would lead to the shortening of the long-term survival of patients. Three randomized controlled trials (RCTs) (2, 29, 30) from China, South Korea, and Japan confirmed the safety and superiority of laparoscopic surgery compared to open surgery for the treatment of AGC, accompanied by additional advantages of no increase in surgical complications and weakening of the prognosis. Significantly, this therapeutic approach can realize less intraoperative bleeding, lower postoperative pain score, shorter length of stay in the hospital, and faster postoperative recovery. Moreover, previous clinical studies have also verified the safety and effectiveness of laparoscopic surgery after NAC for AGC, with an incidence of postoperative complications ranging from 10% to 33.3% (3, 4, 24, 31, 32). Whether the advantage of laparoscopic surgery will continue in NAI combined with chemotherapy aroused tremendous concern. In the current study, the overall complication rate of the NAI group and NAC group was both 30.0%, and grades 3/4 complications in the two groups were two cases (6.7%) and four cases (8.0%), respectively. Similarly, no difference was observed in postoperative complications of NAI plus laparoscopic gastrectomy in our study compared to that of NAC plus laparoscopic gastrectomy and laparoscopic gastrectomy alone reported by previous trials (2, 3). The observed anastomotic leakage in the NAI cohort was lower than the NAC cohort, yet without statistical difference. In these patients, the tumor was located at the proximal stomach and all of them underwent total gastrectomy. Local tissue edema caused by incomplete stricture of the cardia and the high site of anastomosis might be the risk factors. In particular, all of these patients were insensitive to NAC. Moreover, the incidence of postoperative pulmonary infection in the two cohorts was higher (20.0% for NAI and 14.0% for NAC) than that reported previously (3). It may be related to the higher proportion of total gastrectomy in our study (53.3% for NAI and 56.0% for NAC) and regular chest x-ray examination 2 days postoperatively, which may facilitate the detection of mild or asymptomatic pneumonia. It is also correlated with the tendency of pulmonary inflammatory injury after immunotherapy (9), which requires further research to support these speculations. No patients in the two groups converted to laparotomy, and no difference was observed in intraoperative bleeding, operation time, and postoperative gastrointestinal function recovery.

The reported objective response rate of single-agent immunotherapy is about 15% regardless of CPS; and for AGC or gastroesophageal junction cancer, immunotherapy combined with chemotherapy or targeted therapy can significantly improve the objective response rate from 24% to 65.8%, without any increase in the incidence of adverse effects, providing a potential direction of combined treatment of immunotherapy and other regimens for the treatment of AGC (5, 16, 33). In our study, the complete or partial response rate of the NAI group was significantly higher than that in the NAC group, leading to a larger proportion of ypT0 populations. The multivariable model estimated that NAI was as an independent protective factor for the overall response rate. These results may suggest that laparoscopic surgery for AGC after NAI combined with chemotherapy could achieve a better short-term outcome. In our univariable and multivariable analyses for odds to the overall response rate of the NAI group, preoperative CPS and the status of immune cells such as white blood cells and granulocytes failed to be estimated as significant predictors. These may indicate that patients with AGC would also benefit from preoperative immunotherapy when the CPS is negative or suffer from decreased peripheral blood cells. Moreover, the data in our study indicated that patients who are Epstein–Barr virus-encoded RNA-1 (EBER)-positive (10.0%) and with microsatellite instability-high (MSI-H) (10.0%) in the NAI group all obtained partial response or complete response, which shows that a positive relationship of the EBER and MSI status correlates with the efficacy of immunotherapy. Further large-scale trial is needed to confirm the results.

However, our study has the following three main limitations. Firstly, we failed to calculate the sample size of the two groups due to insufficient data on complications after NAI of AGC plus laparoscopic surgery. The application of NAI for the treatment of AGC is a new effort and is exploratory. Our study finds that NAI combined with chemotherapy plus laparoscopic surgery for AGC can obtain a better overall response rate, which might guide later research hypotheses or clinical practice. Secondly, the inconsistencies in chemotherapy regimens may lead to different results within the group. Generally, 5-fluorouracil-based chemotherapy regimens, such as CapeOX, FOLFOX, and FLOT, are considered effective treatments for AGC. The proportion of the CapeOX regimen in the NAC group is lower than that in the NAI group (24.0% vs. 66.7%). This is partially caused by patients in the NAC group having a larger proportion of upper tumor location and incomplete esophageal obstruction. These patients were evaluated by multidiscipline discussion and recommended to undergo intravenous chemotherapy. Thirdly, it was a single-center study, and laparoscopic surgeries reported in our study were conducted by experienced chief surgeons. It may not fully reflect the generality of the experience and skills of surgeons at all levels.

## Conclusion

NAI combined with chemotherapy plus laparoscopic surgery may be a safe therapeutic option for AGC. The NAI regimen exhibits superiority in achieving a better overall response rate, yet without differences in postoperative complications, adverse effects, and postoperative recovery when compared with the NAC regimen. Moreover, findings in our study remain to be confirmed through further multicenter, large-scale, and phase III RCTs.

## Data availability statement

The original contributions presented in the study are included in the article/**Supplementary Material**. Further inquiries can be directed to the corresponding author.

## Ethics statement

The studies involving human participants were reviewed and approved by Ethics committee of Nanfang Hospital Southern Medical University. The patients/participants provided their written informed consent to participate in this study.

## Author contributions

Study conception and design: JS, WG, and YH. Acquisition, analysis, or interpretation of data: All authors. Drafting of the manuscript: JS and WG. Critical revision of the manuscript for important intellectual content: JS, WG, and YH. Statistical analysis: JS, ZC, LW, XM, HH, and YH. Administrative, technical, or material support: HL, LZ, TL, FL, JY, and GL. All authors read and approved the final manuscript.
