# Supplementary material for: Safety and short-term outcomes of laparoscopic surgery for advanced gastric cancer after neoadjuvant immunotherapy: A retrospective cohort study
Source: Front Immunol. 2022 Dec 8;13:1078196. doi: 10.3389/fimmu.2022.1078196 (PMC9779926; doi:10.3389/fimmu.2022.1078196)
Supplement: Supplementary file 1 [file Table_1.pdf]

SUPPLEMENTARY TABLE 1 Clinical data characteristics of as-treat patients

**Variable**	**NAC (n = 50)**	**NAI (n = 30)**	P^b
Sex			0.286
Male	40 (80.0)	20 (66.7)	
Female	10 (20.0)	10 (33.3)	
Age (years)			0.717
Mean[a]	55.5 (11.1)	54.9 (13.2)	
<60	32 (64.0)	17 (56.7)	
60-69	15 (30.0)	10 (33.3)	
≥70	3 (6.0)	3 (10.0)	
BMI (kg/m^2)			0.297[c]
Mean[a]	22.7 (2.7)	23.5 (3.6)	
Range	15.4-27.7	16.6-29.7	
ECOG			1.000
0	12 (24.0)	7 (23.3)	
1	38 (76.0)	23 (76.7)	
cT stage			0.298
T1-2	3 (6.0)	0	
T3	11 (22.0)	4 (13.3)	
T4A	29 (58.0)	18 (60.0)	
T4B	7 (14.0)	8 (26.7)	
cN stage			1.000
N0	4 (8.0)	2 (6.7)	
N+	46 (92.0)	28 (93.3)	
cM stage			0.164
M0	46 (92.0)	24 (80.0)	
M1	4 (8.0)	6 (20.0)	
cTNM stage			0.195
II	7 (14.0)	1 (3.3)	
III	32 (64.0)	17 (56.7)	
IVA	7 (14.0)	6 (20.0)	
IVB	4 (8.0)	6 (20.0)	

Values in parentheses are percentages. [a]Values are shown as mean (SD). [b]*P* value was calculated by $\chi 2$ test or Fisher's exact test, except for [c]Mann-Whitney test.

Abbreviations: NAI, neoadjuvant immunotherapy; NAC, neoadjuvant chemotherapy.

SUPPLEMENTARY TABLE 2 Postoperative complications comparison with former studies.

Variable	NAI+LG (n = 30)	NAC+LG (n = 45)	LG (n = 519)	*P* value[b]
Clavien Dindo classification				
Grade I	0	0	7 (1.3)	1.000
Grade II	8 (26.7)	6 (13.3)	54 (10.4)	0.028
Grade IIIa	0	4 (8.9)	12 (2.3)	0.053
Grade IIIb	1 (3.3)	1 (2.2)	3 (0.6)	0.122
Grade IVa	1 (3.3)	1 (2.2)	1 (0.2)	0.043
Grade IVb	0	0	0	NA
Grade V	0	0	2 (0.4)	1.000
Overall complications[a]	9 (30.0)	9(20.0)	79 (15.2)	0.083

Values in parentheses are percentages. LG, laparoscopic gastrectomy; NA, not applicable; NAC, neoadjuvant chemotherapy; NAI, neoadjuvant immunotherapy. [a]Multiple complications may occur in one patient. [b]*P* value was calculated by Fisher's exact test.
